# Usability Study of the iACTwithPain Platform: An Online Acceptance and Commitment Therapy and Compassion-Based Intervention for Chronic Pain

**DOI:** 10.3389/fpsyg.2022.848590

**Published:** 2022-07-22

**Authors:** Raquel Guiomar, Inês A. Trindade, Sérgio A. Carvalho, Paulo Menezes, Bruno Patrão, Maria Rita Nogueira, Teresa Lapa, Joana Duarte, José Pinto-Gouveia, Paula Castilho

**Affiliations:** ^1^University of Coimbra, Center for Research in Neuropsychology and Cognitive Behavioral Intervention, Faculty of Psychology and Educational Sciences, Coimbra, Portugal; ^2^Digital Human-Environment Interaction Lab, School of Psychology and Life Sciences, Lusófona University, Lisbon, Portugal; ^3^University of Coimbra, Institute of Systems and Robotics, Coimbra, Portugal; ^4^University of Coimbra, Department of Electrical and Computer Engineering, Coimbra, Portugal; ^5^Pain Unit, Coimbra Hospital and University Centre, Coimbra, Portugal; ^6^Faculty of Health Sciences, University of Beira Interior, Covilhã, Portugal; ^7^Department of Psychology, Lund University, Lund, Sweden

**Keywords:** acceptance and commitment therapy, chronic pain, online intervention, usability study, IT

## Abstract

**Background:**

This pilot study aims to test the usability of the iACTwithPain platform, an online ACT-based intervention for people with chronic pain, to obtain information on which intervention and usability aspects need improvement and on expected retention rates.

**Methods:**

Seventy-three Portuguese women with chronic pain were invited to complete the first three sessions of the iACTwithPain intervention assess their quality, usefulness and the platform’s usability. Twenty-one accepted the invitation. Additionally, eight healthcare professionals working with chronic medical conditions assessed the platform and the intervention from a practitioner’s point of view.

**Results:**

This study presented a considerable attrition rate (71.43%) among chronic pain participants, with six completers. There were no significant differences in demographic or clinical variables between dropouts and completers except for completed education (participants who dropped out presented less education than completers). Reasons for dropout were related to difficult personal events occurring during the time of the intervention, lack of time, or having forgotten. There seemed to be an overall satisfaction with both the intervention, its contents and form of presentation of information, and the platform, concerning its design, appearance, and usability. Real image videos were preferred over animations or audio by chronic pain participants. Healthcare professionals emphasized the appealing and dynamic aspects of the animation format.

**Conclusion:**

This study informs the ongoing improvement of the iACTwithPain platform and provides valuable information on aspects researchers should consider while developing online psychological interventions for chronic pain. Further implications are discussed.

## Introduction

Chronic pain can be defined as persistent pain lasting more than 3 months, has a prevalence of approximately 20% in adults (Elliott et al., 1999), can have a detrimental impact in mobility and quality of life (Smith et al., 2001), and productivity (Dorner et al., 2016). Chronic pain is associated with high economic burden, and is considered as one of the most expensive long-term health conditions in industrialized countries (Bernfort et al., 2015; Groenewald and Palermo, 2015; Mayer et al., 2019).

Acceptance of pain is considered an important factor for a successful adaptation to chronic pain, linked to less depression, pain-related anxiety, and disability (McCracken and Eccleston, 2003). These findings have supported the application of acceptance-based therapies, such as Acceptance and Commitment Therapy [ACT; Hayes et al. (2012)], to this population. ACT is an empirically validated psychological approach for chronic pain (APA Presidential Task Force on Evidence-Based Practice, 2006) that promotes acceptance and engagement with values-guided behavior despite chronic pain symptoms (Vowles and McCracken, 2008). The efficacy of ACT for chronic pain has been demonstrated in a meta-analysis by Veehof et al. (2011), and in a broader review including both acceptance and mindfulness-based interventions (Veehof et al., 2016). At the same time, the pertinence of promoting self-compassion [i.e., the ability to be sensitive to personal suffering and being motivated to kindly alleviate it; (Neff, 2003; Gilbert, 2009)] in chronic pain has been highlighted due to its protective role against depressive symptomatology (Carvalho et al., 2018) and the positive effects compassion-focused interventions have presented in this population (Gooding et al., 2020). The combination of ACT and compassion for chronic pain was recently implemented in a pilot test with promising results (Carvalho et al., 2021a).

In recent years, online-delivered interventions have generated increased interest due to their accessibility, flexibility and cost-effectiveness (Bergmo, 2015). In particular, online-based ACT has been proved to be efficacious for chronic pain in a recent meta-analysis. Online-ACT was greater than control conditions in reducing pain interference, pain intensity, depression, and anxiety, and in increasing mindfulness, and psychological flexibility (Trindade et al., 2021a). For these reasons, and additionally considering the advantages of online interventions to improve health-related outcomes (Bergmo, 2015), and for chronic pain in particular (Trindade et al., 2021a), the iACTwithPain intervention platform was developed by the authors of this paper (iACTwithPain research team). iACTwithPain is an ACT- and Compassion-based intervention tailored explicitly to chronic pain. It comprises eight sessions to be completed on an online platform throughout 8 weeks (1 session per week). The efficacy of the iACTwithPain intervention in improving chronic pain impact and related health and quality of life markers will be tested in full in a randomized controlled trial (Carvalho et al., 2021b). It is the aim of the current study to pilot test the usability of the platform by examining the feedback of clinicians and chronic pain patients of first three sessions in order to obtain information on which intervention and usability aspects need improvement, and on expected retention rates. Given our team’s combined expertise in psychology, design and engineering, we hypothesize qualitative feedback from participants reflecting pertinent content for patients with chronic pain, and an online platform that is intuitive, engaging and esthetically pleasing. We additionally expect high usability scores, as measured by the System Usability Scale [SUS; Martins et al. (2015)], relating to participants’ use of the platform.

## Methods

### Ethical Approval and Data Safety

This study was approved by Ethical Committee of the Faculty of Psychology and Educational Sciences of the University of Coimbra (on 28/11/2019), and was conducted in accordance with the ethical standards in the 1964 Declaration of Helsinki and its later amendments. All collected data will be stored (for 5 years) using high standard security mechanisms, and thus ensuring confidentiality. Data will be in anonymized and can only be assessed by the research team.

### Sample Size Calculation

According to Faulkner (2003), on average, a sample of five participants can detect 85% of the usability problems. Therefore, we aim to have a sample of at least 5 in each group (participants with chronic pain and health professionals). Assuming a conservative dropout rate of 83% (Bangor et al., 2009) for the clinical sample, at least 30 participants should be invited to enroll in the study.

### Procedures

The study’s chronological order was as follows: (1) participants recruitment (presentation of the study and informed consent); (2) during the following 2 weeks participants tested the iACTwithPain platform; (3) in the third week the usability and quality assessment questionnaire was administered; (4) participants who dropped out from the study were contacted to fill in a questionnaire (reasons for dropping out).

Seventy-three Portuguese women with chronic pain, enrolled in a different study who had demonstrated interest in taking part in the current one, were invited to complete the first three sessions of the iACTwithPain intervention and assess their quality and usefulness, as well as to assess the platform’s usability. Inclusion criteria were: age between 18 and 65 years; diagnosis of chronic pain; internet access; and proficiency in Portuguese. Exclusion criteria were: not providing informed consent; or pain due to malignancy, trauma, or surgery. Twenty-one accepted the invitation to participate, signed an informed consent, and were enrolled in the study. Participants with chronic pain that did not complete the three sessions were asked to fill out a questionnaire on the reasons for attrition.

Additionally, nine healthcare professionals working with chronic medical conditions (four psychologists, four physicians, and one nurse) were also invited to assess the platform and the intervention from a practitioner’s point of view. Of these, eight signed an informed consent before the start of the study and were enrolled in the study.

### The iACTwithPain Platform

The iACTwithPain intervention was designed based on the psychological flexibility model (Vowles and McCracken, 2008; Trindade et al., 2020) and compassion-focused interventions applied to chronic pain (Carvalho et al., 2018). The psychologists’ members of the iACTwithPain team have expertise in developing and efficacy testing ACT and compassion-based interventions for chronic conditions such as chronic pain (Carvalho et al., 2021b), cancer (Trindade et al., 2020), inflammatory bowel disease (Trindade et al., 2021b), and psychiatric disorders (Duarte et al., 2017). Moreover, the team’s knowledge of the psychological impact of chronic pain and the underlying psychological processes (Carvalho et al., 2018, 2019, 2021c) were taken into consideration. During iACTwithPain development, the principles of ACT and compassion-based interventions were strictly followed to ensure pertinent and rigorous therapeutic sessions. As described in the RCT protocol (Carvalho et al., 2021b) treatment integrity guidelines for ACT (Plumb and Vilardaga, 2010) were followed and included: (a) training in ACT and compassion focused therapy models, ensuring that the therapists fully grasp the concepts and principles of the interventions and have previous competence/experience in their application; (b) the content of the sessions are ACT-consistent (for example focus on the function rather than the content); and the known processes of change of ACT and compassion were followed during the development of the intervention.

Participants will be randomly assigned to two experimental arms: and ACT-only intervention or an ACT and compassion-focused intervention. All sessions will be the same except for sessions five and six. The ACT and compassion-focused group will be exposed to compassion themes and exercises (e.g., what is compassion, developing compassion toward the self and others, obstacles to compassion). The ACT-only group will reinforce previous topics (e.g., willingness, acceptance, defusion, and observing self) without introducing new information or practices. The ACT intervention will include the following core themes: (a) Awareness of internal experiences (mindfulness; self-as-context); (b) Openness to experience difficult experiences (acceptance of pain: willingness toward thoughts, emotions and physical sensations; and cognitive defusion); (c) Engagement with valued action (values clarification and commitment to meaningful actions); (d) Self-Compassion (promoting self-kindness in times of difficulty; for the ACT and compassion-focused group only). Sessions will comprise video-animations (with videoscribes and therapists’ avatars), real-image videos, texts, and audios guiding meditative practices. iACTwithPain is a self-paced intervention and therefore the participant can decide when to login and complete each session. Nevertheless, the intervention was designed so that sessions are completed weekly (one session per week, over 8 weeks). Each new session will become available in the platform every Monday. Emails notifying the participants that a new session is available will be sent weekly.

**FIGURE 1:**
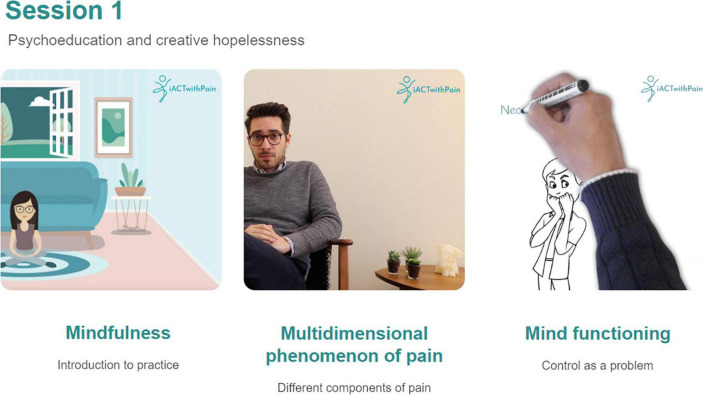
Example of session 1 contents depicting the three types of videos (left to right: video-animation with a therapist avatar, real-image video with a therapist, and videoscribe animated video).

Session 1 is completed immediately after session 0, and the following sessions are then completed once per week. A brief introductory session (session 0) will welcome participants to the intervention and introduce the platform. Participants are asked to practice between-session mindfulness and/or compassion-based meditative exercises as often as possible. In this usability study, participants were asked to complete the introductory session (session zero), session one and two. Session one focuses on psychoeducation about chronic pain, promotion of creative hopelessness, introduction to mindfulness practice, and mindfulness of breathing practice. Session two focuses on the usefulness of mindfulness to manage suffering, promotion of mindfulness practice, and body scan practice (see Table 1 for more detailed information). Mindfulness exercises throughout the intervention focus on a non-judgmental, open, and accepting attitude toward the present moment. Visual examples from these sessions are presented in Figure 1.

**TABLE 1:** Structure and contents of session 0, 1, and 2.

Topic	Content/Exercise	Format
**Session 0 –** Introduction to the intervention and the platform		
Welcome video	Information about the nature and structure of the program, and its objectives.	Real-image video with a therapist
Navigation of the platform	Presentation of the platform and information regarding its navigation (including instructions and steps to move through the program)	Real-image video
Motivation and intentions clarification	Contemplative exercise “Exploring my motivations to do this intervention”	Real-image video with a therapist
**Session 1 –** Psychoeducation and creative hopelessness		
Check-in	Brief exercise to focus and anchor on the present moment (a soft landing exercise).	Video-animation with a therapist avatar
Psycho-education about chronic pain	Multidimensional phenomenon of pain: video about the function of pain, how it manifests itself in the body, and its various components.	Real-image video with a therapist
The problem with our problem-solving minds (controlling is the problem)	Video about how the human mind works and how it attempts to control unpleasant internal experiences, and consequently generating suffering and exacerbating our problems.	Videoscribe animated video
Promotion of creative hopelessness	Video about exploring alternative ways of relating to our sensations, thoughts, memories and emotions. One of the core competencies that will be developed in the iACTwithPain program is the ability to be in the present moment. This competence will be developed through mindfulness training.	Real-image video with a therapist
Introduction to mindfulness practice	Instructions on proper posture to perform contemplative practices (appropriate body postures for the contemplative practices are exemplified).	Real-image video with a therapist
	Mindfulness of breathing practice.	Audio
Between-session assignment	Mindfulness of breathing practice.	Audio
**Session 2 –** The body as an anchor on the present moment		
Check-in	Brief exercise to focus and anchor on the present moment (a soft landing exercise).	Video-animation with a therapist avatar
Mindfulness as a key aspect to manage suffering	Video presenting what Mindfulness is and its benefits.	Real-image video with a therapist
Therapists’ personal experience with mindfulness: Tips for maintaining regular practice	In this video, therapists share their personal experience with mindfulness. Not only the difficulties experienced, but also the benefits obtained, resulting from a frequent and committed practice.	Real-image video with a therapist
The body as an anchor to the present moment	In our body everything is integrated. All systems, organs, muscles and nerve endings are interconnected and communicate with each other. Our emotions also manifest in the body (for example when we are anxious, we may feel tension in the muscles or the heart beating faster). Video about the complex entity that is the body, and exploration of usual and alternative ways of relating to our emotions, thoughts, and feelings, as inhabitants of the body.	Videoscribe animated video
Between-session assignment	Mindful movement (Qigong)	Real-image video with a therapist
	Body scan meditation	Audio

*Information partly retrieved from Carvalho et al. (2021b).*

### Measures

#### Primary Outcome

Both groups of participants (patients and professionals) were asked to complete the SUS [(Brooke, 1996); original validation (Bangor et al., 2008); portuguese validation (Martins et al., 2015)]. This scale is robust and widely used to evaluate the usability of products and user interfaces (Bangor et al., 2009). The European Portuguese validation is equivalent to the original version in terms of semantics and content, and presented good psychometric properties e.g., high convergent validity and satisfactory inter-rater percentage of agreement (Martins et al., 2015). It provides a single reference score for participants’ view of the usability of a product or service. It comprises ten items rated on a 5-point Likert scale (1: Strongly Disagree; 5: Strongly Agree) and has been shown to present adequate psychometric properties (Bangor et al., 2008). The SUS items were coded by subtracting 1 from the odd items’ score, subtracting even items’ score from 5 (correction for the reverse scored items), and multiplying the re-coded values by 2,5 (Martins et al., 2015). This results in new scores ranging from 0 to 100, where higher scores indicate better usability. According to the adjective ratings proposed by Bangor et al. (2008), SUS scores from 0 to 25 are “Worst imaginable,” 25–39 “Poor,” 39–52 “Ok,” 52–73 “Good,” 73–85 “Excellent,” and 85–100 are “Best imaginable” (Bangor et al., 2008).

#### Secondary Outcomes

Participants with chronic pain and healthcare professionals were asked to complete a set of self-report questions regarding the quality of the intervention (e.g., content clarity, pertinence of the intervention themes, platform design and organization, individual session quality), session duration, and preferences on type of presentation format (e.g., video, audio). These questions were developed by the research team to tailor to the specific platform characteristics that this study aims to assess. The platform kept track of participant engagement concerning the number of logins in the platform and engagement with mindfulness practice audios or videos.

### Statistical Analyses

All analysis were performed on SPSS (Statistical Package for the Social Sciences), version 25 (IBM Corp., Armonk, NY, United States). Descriptive and frequency analyses were conducted to analyze participants’ evaluations of the platform/intervention, as well as engagement with the platform. Mann–Whitney U and Fisher’s exact test were conducted to analyze differences between groups of participants (e.g., completers, dropouts). The retention rate was computed as the proportion of participants that completed the three sessions.

## Results

In this study we aimed to pilot test the usability of the iACTwithPain platform, in what concerns our primary outcome – usability scores; and secondary outcomes – qualitative assessment, engagement, and retention rate.

### Primary Outcome – Usability

#### Sample of Participants With Chronic Pain

Regarding usability scores (SUS), on average, iACTwithPain’s platform was rated as excellent (*N* = 5, *M* = 76.50, SD = 16.83). Two participants rated it as good (*M* = 58.75, SD = 1.77), one rated it as excellent (82.5), and two rated it as best imaginable (*M* = 91.25, SD = 5.30).

#### Sample of Healthcare Professionals

On average, the iACTwithPain’s platform usability (SUS) was rated by healthcare professionals as excellent (*N* = 5, *M* = 84.50, SD = 7.79). Two participants rated it as excellent (*M* = 80.00; SD = 2.50), and three as best imaginable (*M* = 91.25; SD = 8.84).

### Secondary Outcomes – Qualitative Assessment, Engagement, and Retention Rate

#### Qualitative Assessment of the iACTwithPain Platform

##### Sample of Participants With Chronic Pain

Of the six completers, three preferred real image video format, two preferred animations, and one preferred sole audios. Table 2 presents participants’ feedback on each of these formats. Written feedback highlighted advantages to the real image video format, which was overall described as attractive and motivating to practice. One comment indicated that more investment in the background of the real-time videos should be made and that these videos could include animations. Described advantages for the audio format were accessibility and having less distractions. Animation videos were described as interesting and having an attractive and simple design.

**TABLE 2:** Participants’ feedback on the different kinds of content format (*n* = 6).

Questions	Participants endorsing each response category, *n*					*M* (SD)
	1. Strongly disagree	2. Slightly disagree	3. Neither disagree nor agree	4. Slightly agree	5. Strongly agree	
**Audio**						34.16
Easy to use	0	0	0	2	4	4.67 (0.52)
I got easily distracted	1	1	0	3	1	3.33 (1.52)
Interesting	0	0	0	4	2	4.33 (0.52)
I would use this format again	0	0	0	4	2	4.33 (0.52)
Appropriate lenght	0	1	0	3	2	4.00 (1.10)
Has technical quality	0	2	0	2	2	3.67 (1.37)
Boring and uninteresting	4	1	0	1	0	1.67 (1.21)
Message is clear and easy to understand (*)	0	0	0	2	3	4.60 (0.55)
Appealing and increases motivation	0	0	0	4	2	4.33 (0.52)
**Animation**						35.60
Easy to use (*)	0	0	0	3	2	4.40 (0.55)
I got easily distracted (*)	2	2	1	0	0	1.80 (0.84)
Interesting (*)	0	0	0	2	3	4.60 (0.55)
I would use this format again (*)	0	0	0	2	3	4.60 (0.55)
Appropriate lenght (*)	0	0	0	3	2	4.40 (0.55)
Has technical quality (*)	0	0	0	1	4	4.80 (0.45)
Boring and uninteresting (*)	4	0	0	1	0	1.60 (1.34)
Message is clear and easy to understand (*)	0	0	0	1	4	4.80 (0.45)
Appealing and increases motivation (*)	0	0	0	2	3	4.60 (0.55)
**Real image video**						36.66
Easy to use	0	0	0	1	5	4.83 (0.41)
I got easily distracted	2	1	1	2	0	2.50 (1.38)
Interesting	0	0	0	2	4	4.67 (0.52)
I would use this format again	0	0	0	2	4	4.67 (0.52)
Appropriate lenght	0	0	0	2	4	4.67 (0.52)
Has technical quality	0	0	1	1	4	4.50 (0.84)
Boring and uninteresting	4	1	0	1	0	1.67 (1.21)
Message is clear and easy to understand	0	0	0	2	4	4.67 (0.52)
Appealing and increases motivation	0	0	1	1	4	4.50 (0.84)

(*) one participant did not respond to these topics.

Concerning other aspects of the intervention and platform, participants generally rated positively their satisfaction (on a scale from 0, no satisfaction, and 10, extremely satisfied) with the platform’s design (*M* = 7.50; SD = 2.01), color pattern (*M* = 8.33; SD = 1.37), and attractiveness (*M* = 7.67; SD = 1.75).

The topics covered by the two assessed sessions were very positively evaluated (*M* = 8.67; SD = 1.03). The topics found most useful to participants (*n* = 4) were psychoeducation about pain (*n* = 1), mindfulness of breathing (*n* = 1), and body scan practice (*n* = 2). Participants also provided positive answers (on a scale from 0, no satisfaction, and 10, extremely satisfied) regarding their interest in continuing the intervention (*M* = 7.33; SD = 3.20). Furthermore, four participants provided feedback on what they believe could be changed to improve these three sessions. One participant indicated that the sessions seemed to cover all important aspects. Another referred to longer intervals without instructions in the mindfulness practices, and two stated that they felt that strategies to reduce pain should be included in the intervention.

##### Sample of Healthcare Professionals

The healthcare professionals group seemed to prefer the animation (*n* = 3) and real image video (*n* = 2) formats. Real image videos (*M* = 34.20; SD = 2.68) and animations (*M* = 33.60; SD = 1.67) were overall more positively evaluated than audios (*M* = 27.80; SD = 3.49). Overall, written feedback about the audios indicated that this format is easy to use and accessible, although not particularly appealing or stimulating. Animations were described as attractive, and appealing, but impersonal. Real image videos were described as providing a connection with the therapeutic team and the opportunity to get to know the therapists better. Some technical issues were also identified regarding this format (e.g., indications to improve the flow of the video cuts; audio and video synchronization).

Healthcare professionals presented favorable ratings on other aspects of the platform (on a scale from 0, no satisfaction, and 10, extremely satisfied): design (*M* = 8.40; SD = 1.52), color pattern (*M* = 8.80; SD = 1.30), and attractiveness (*M* = 8.40; SD = 1.34).

Concerning the contents of the intervention, positive ratings were also provided (0, no satisfaction – 10, extremely satisfied) for all assessed items: content quality (*M* = 9.60; SD = 0.55), pertinence of the topics (*M* = 9.40; SD = 0.89), topics sequencing (*M* = 8.60; SD = 0.55), and appropriateness of therapists’ posture (*M* = 9.20; SD = 0.84).

#### Participant Engagement and Retention

The completers’ sample logged into the platform on average 16.33 times (SD = 12.91), and evaluated the first session on average as 4 (SD = 0.63), and the second session as 3.83 (SD = 0.75), both on a scale of 1–5. In what concerns home practice, completers practiced on average 2.67 times (SD = 3.20) the practice from session one (mindfulness of breathing), and 2.20 times (SD = 2.68) the practice from session 2 (body scan).

The dropout sample logged into the platform on average 3.33 times (SD = 3.90). Four of these participants completed the first session, assessed it as 4.5 on average (from 1 to 5; SD = 0.58), and did the practice from session one on average 1.75 times (SD = 0.96). No participant from the dropout group assessed the second session nor engaged in the second practice.

#### Reasons for Dropout

Six of the 12 participants that dropped out from the study completed the attrition questionnaire. Three participants indicated a difficult personal situation after the beginning of the study as the reason for dropout, two referred to lack of time, and one referred to having forgotten to complete the intervention (although participants were reminded once a week to complete the sessions). No participant referred to any reason associated with the platform or the intervention. Two participants provided additional feedback, indicating that: (1) “it looked very well structured, simple, and potentially very useful”; and (2) “I really liked the intervention’s contents and how the platform was structured. I had a problem with the login once, but it was quickly fixed.”

#### Descriptive Statistics

##### Sample of Participants With Chronic Pain

The demographic and clinical characteristics of the participants that accepted to participate, the ones who dropped out, and the completers are presented in Table 3.

**TABLE 3:** Descriptives and frequencies of the demographic and clinical variables in study across groups, and tests of differences between participants who dropped out and completers.

		Accepted to participate (*N* = 21)	Dropped-out (*N* = 12)	Completers (*N* = 6)	Test of differences		
					Mann Whitney U	Fisher’s exact test	*p*
Age, *M* (SD)		45.35 (6.70)	45.09 (4.01)	46.67 (11.55)	−32.50	–	0.960
	Middle school	1 (4.80)	1 (8.30)	–		10.32	0.007
	High school	8 (38.10)	4 (41.70)	–			
Education, *n* (%)	BSc degree	6 (28.60)	2 (16.70)	4 (66.70)	–		
	Post-graduation	4 (19.00)	4 (33.3)	–			
	MSc degree	2 (9.50)	–	2 (33.3)			
	1–5 years	5 (23.80)	1 (8.30)	3 (50)	–	3.65	0.177
Time since diagnosis, *n* (%)	5–10 years	4 (19.00)	3 (25.00)	1 (16.70)			
	10+ years	12 (57.10)	8 (66.70)	2 (33.30)			
	Fibromyalgia	16 (76.20)	9 (75.00)	4 (66.70)	–	3.42	0.769
Chronic pain diagnosis, *n* (%)	Rheumatoid arthritis	2 (9.50)	1 (8.30)	1 (16.70)			
	Sjogren Syndrome	1 (4.80)	1 (8.30)	0 (0)			
	Low back pain	1 (4.80)	0 (0)	1 (16.70)			
	Scleroderma	1 (4.80)	1 (8.30)	0 (0)			
Comorbid medical condition diagnosis, *n* (%)	Yes	11 (52.40)	5 (41.70)	4 (66.70)		–	0.620
	No	10 (47.60)	7 (58.30)	2 (33.30)			

*M, mean; SD, standard deviation.*

There were no statistically significant differences between completers and participants who dropped out regarding age, time since chronic pain diagnosis, chronic pain diagnosis, and diagnosis of comorbid medical conditions. However, there were significant differences regarding education - participants that dropped out presented less education. The final sample of completers was composed of women with chronic pain with ages between 35 and 65 years.

##### Sample of Healthcare Professionals

Regarding the group of healthcare professionals, out of the eight professionals that signed an informed consent, five (four psychologists and a physician) provided feedback. These five professionals, four women and a man, had worked in chronic pain contexts between 5 and 20 years (*M* = 10.40; SD = 6.19) in hospitals in the North and Center regions of Portugal.

## Discussion

Overall, the pilot usability study of the iACTwithPain platform (and first three sessions: 0, 1, and 2) presented promising results in terms of usability scores (high for both clinical and healthcare professionals samples), quality assessment of the different content delivery methods, and engagement metrics. Qualitative feedback from participants and health professionals will be taken into consideration for the development of the full iACT intervention, and measures to avoid dropout will be adopted.

Of the six chronic pain participants that completed this pilot test, and the five healthcare professionals that provided feedback, there seemed to be an overall satisfaction with both the intervention, regarding its contents and form of presentation of information, and the platform, concerning its design, appearance, and usability. Real image videos, mainly used to introduce new topics, provide rationales, or exemplify the possible meditation positions, appeared to be more preferred than animations or audios by chronic pain participants. This was possibly due to a perception of “being closer” to the therapists, provided by the real image videos, which may help participants feel understood and find the motivation to practice. It may also be hypothesized that real image videos are more effective in tapping into tacit cognitive-emotional factors of efficacy in psychotherapy, such as an empathic and compassionate therapeutic relationship, which is more difficult to convey through non-human avatars. This was highlighted by healthcare professionals’ feedback, who nonetheless also emphasized the appealing and dynamic aspects of the animation format. However, for meditative practices, the audio format should have preferred use over real image videos or animations to avoid unnecessary distractions during practice. Finally, two participants with chronic pain indicated that they believed that strategies to reduce pain should be included in the intervention. Even though the first session of the iACTwithPain intervention states the rationale for accepting pain rather than attempting to reduce or control it (Vowles and McCracken, 2008), and the negative effects of doing the latter (McCracken et al., 2007), it seems that these participants still held on to the idea that pain must be avoided to lead a satisfactory life. Still, participants had only completed the two first sessions of the intervention, where ACT’s acceptance and values topics are not clearly focused on (these topics are presented more in-depth in later sessions). Therefore, it might be beneficial for the future RCTs to increase the focus on acceptance earlier in the intervention, by including, for example, experiential acceptance exercises or metaphors.

The study attrition rate was of 71.43%. Out of the 21 participants that signed the informed consent, 12 (57.14%) accessed the intervention, and 6 (28.57%) completed the study. No significant differences in demographic or clinical variables were found between dropouts and completers except for completed education, with participants who dropped out presenting less education than completers. This result is in line with findings from previous research, where lower educational level was associated with higher risk if dropping out (Karyotaki et al., 2015). Motives for dropout, offered by 6 of the 12 participants who did not finish the intervention, were unrelated to the intervention or platform. There were reasons related to difficult personal events occurring during the intervention, lack of time, or having forgotten. Similar results were found in a review about reasons for dropping out in ACT interventions (Karekla et al., 2019). The iACTwithPain platform will thus include mechanisms to identify when participants have not logged in to the platform for 3 days in a row, so the team can send engagement reminders to these participants. This strategy is aligned with previous studies showing that contingent reminders can improve motivation and boost behavior change (Webb et al., 2010). If emails are not enough to engage non-responsive participants, phone calls will be implemented, since previous online-based studies have suggested that closer contact with the therapist is associated with lower dropout rates (Cuijpers et al., 2008; Macea et al., 2010). Still, given that only half of the participants who dropped out provided reasons for having stopped participating, it is difficult to know whether the platform/intervention did not influence some participants’ dropout. Other factors that we did not account for might be influencing dropout, namely comorbid depression or anxiety, relationship status, or chronic pain severity, according to a review by Melville et al. (2010). The attrition rate presented by this study falls within the range of 2–83%, presented by a review on dropout rates from online treatments for psychological disorders (Melville et al., 2010). It should nonetheless be noted that the current intervention is directed at people with chronic pain, and most importantly, that this is a usability study, in which participants may present less motivation to participate in comparison with a trial of a full intervention.

This usability study will improve the iACTwithPain platform by informing which type of format is preferred and which one works best for each type of content. Considering feedback from the participants, the real image videos with therapists were overall preferred and viewed as engaging and motivating, so we will mostly select this format in the future intervention. Nevertheless, all types of exercises will be maintained (animations, videoscribes, real image videos, and audios), since some participants showed preference for these formats and since this will allow for a more varied and stimulating user experience. The audio format will be selected for guided exercises (e.g., 80th Birthday Party) or meditations (e.g., body scan), since participants reported that this format helped them maintain focus on the instructions of the exercises. Participants did not provide negative feedback regarding the attractiveness, navigation or esthetic elements (e.g., design and color pattern) of the platform. Therefore, no changes will be made to these elements. Finally, weekly automatic and contingent reminders (when a participant does not login in for three consecutive days) will be implemented to reduce the likelihood of participants dropping out from study.

This study has some limitations that are worth discussing. We did not assess previous experience with acceptance or compassion-based psychological interventions, so it was not possible to control the possible confounding effect of previous knowledge/experience on these approaches. The small sample size might have influenced the results obtained and might not generalize to broader samples. Only women enrolled in this study (although this was not a criterion for this study), which limits generalization of these results to male chronic pain samples. However, this usability study allowed the initial test of the iACTwithPain platform and provided valuable insights for developing the full intervention. A future feasibility study informed by the current one is being conducted by the iACTwithPain team and will assess several recruitment pathways/strategies, as well as the full intervention (both quantitatively and qualitatively).

The present study has informed the improvement of the iACTwithPain platform before its final testing in a larger clinical trial. In addition, this study provides useful information on some aspects researchers should consider while developing online psychological interventions for chronic pain.

## Data Availability Statement

The raw data supporting the conclusions of this article will be made available by the authors, without undue reservation.

## Ethics Statement

The studies involving human participants were reviewed and approved by the Faculty of Psychology and Educational Sciences, University of Coimbra, Portugal. The patients/participants provided their written informed consent to participate in this study.

## Author Contributions

IT: conceptualization, data curation, formal analysis, and writing—original draft, review and editing. RG: data curation, formal analysis, and writing—original draft. SC, PM, MN, TL, JD, and JP-G: writing—review and editing. BP: data curation and writing–review and editing. PC: funding acquisition, conceptualization, supervision, and writing—review and editing. All authors read and approved the final manuscript.

## Conflict of Interest

The authors declare that the research was conducted in the absence of any commercial or financial relationships that could be construed as a potential conflict of interest.

## Publisher’s Note

All claims expressed in this article are solely those of the authors and do not necessarily represent those of their affiliated organizations, or those of the publisher, the editors and the reviewers. Any product that may be evaluated in this article, or claim that may be made by its manufacturer, is not guaranteed or endorsed by the publisher.

## Abbreviations

ACT: Acceptance and Commitment Therapy
SUS: System Usability Scale
M: mean
SD: standard deviation.
